# Charles bonnet sydrome: A case report

**DOI:** 10.1192/j.eurpsy.2021.1974

**Published:** 2021-08-13

**Authors:** A. Izquierdo, P. Del Sol Calderon, M. García Moreno

**Affiliations:** 1 Psychiatry, HOSPITAL UNIVERSITARIO PUERTA DE HIERRO MAJADAHONDA, Majadahonda, Spain; 2 Psychiatry, Hospital Universitario Puerta de Hierro, Majadahonda, Spain; 3 Psychiatry, HOSPITAL UNIVERSITARIO PUERTA DE HIERRO MAJADAHONDA, MADRID, Spain

**Keywords:** hallucination, psychosis, Charles Bonnet

## Abstract

**Introduction:**

Faced with recent onset psychotic symptoms in patients over 60 years of age without a psychiatric history, it is important to carry out an adequate differential diagnosis.

**Objectives:**

The objective is to carry out a review of Charles Bonnet syndrome through the presentation of a case

**Methods:**

75-year-old patient who suddenly began to present auditory hallucinations. The patient had no relevant psychiatric history or medical history. She reported that suddenly, two months ago, she had begun to listen to his neighbor through the walls of his home. She heard him talk about her, threatening and insulting her. Later, as a result of these hallucinations, she began to believe that in the bathroom he was spying on her through a camera, forcing her to shower in the dark. Weeks later, she thought that he was also chasing her down the street through a chip that had implanted her. She was distressed and highly anxious. She had started not sleeping out of fear of this neighbor.

**Results:**

In addition to the psychiatric evaluation, an MRI was requested to rule out incipient cognitive deterioration, as well as a hearing examination. It was found that he had severe hearing loss in the left ear. Given these findings, he was diagnosed with Charles Bonnet syndrome.

**Conclusions:**

Charles Bonnet syndrome is normally associated with blindness, however, it is also described in deafness. It occurs with hallucinations of the lost sensory organ. It is a clinical picture that does not respond well to treatments.

**Disclosure:**

No significant relationships.

